# Real‐time anti‐poaching tags could help prevent imminent species extinctions

**DOI:** 10.1111/1365-2664.12452

**Published:** 2015-07-24

**Authors:** Paul O'Donoghue, Christian Rutz

**Affiliations:** ^1^ Faculty of Applied Science University of Chester Chester UK; ^2^ School of Biology University of St Andrews St Andrews UK

**Keywords:** anti‐poaching measures, biologging, elephant, environmental education, illegal trade, ivory, poaching, rhino, tiger, wildlife crime

## Introduction

At an estimated $7–10 billion annually, the global trade in illegal wildlife parts is comparable in economic value to human trafficking, and the smuggling of weapons and drugs (Wasser *et al*. 2008; Wyler & Sheikh 2013). Basic economic principles of supply and demand ensure that, as target species become ever rarer, their market value continues to rise, gradually pushing them towards extinction (Courchamp *et al*. 2006; Nowell 2012a). One particular problem is that anti‐poaching rangers often arrive too late at crime scenes to arrest criminals, making poaching a low‐risk and high‐gains enterprise (Wyler & Sheikh 2013). Here, we identify an opportunity to address this fundamental problem – we propose that cutting‐edge tracking technology could be harnessed to implement effective ‘real‐time poaching‐alert systems’. Animals would be fitted with miniature electronic devices (‘biologgers’) that can detect a poaching event, establish its exact location and relay data remotely to ground teams. Such systems should considerably increase the chances of successful interception, and thereby, escalate the actual and perceived risks of poaching, establishing a powerful new deterrent. In combination with other mitigation strategies (reviewed below), this innovative approach could lead to a much‐needed breakthrough in the increasingly desperate fight against wildlife crime.

## Almost gone

While a wide range of species is targeted for illegal trading, we focus here on the poaching of large mammals, as these are often particularly vulnerable due to their naturally low population densities and reproductive rates. Three case studies serve to illustrate the urgency of implementing effective anti‐poaching measures (cf. Nowell 2012b), but our novel approach would no doubt benefit many other species.

**Figure 1:**
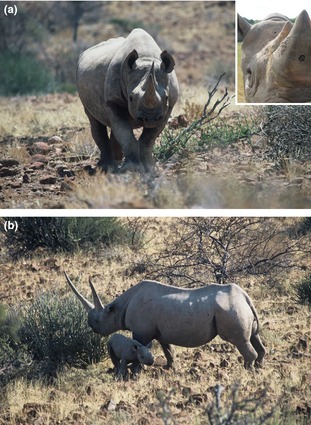
Real‐time poaching‐alert tags could prevent the imminent extinction of rhinos. (a) A black rhino *Diceros bicornis* bull in Damaraland, Namibia, home to one of the last free‐living populations of this critically endangered species; photograph: Tom Collier. Inset: real‐time poaching‐alert tags could be fitted inside rhinos’ horns (cf. Fig. 2). Here, a captive black rhino bull has been fitted with a miniature video camera during pilot trials carried out at Port Lympne Wild Animal Park, Kent, UK; photograph: Paul O'Donoghue. (b) A black rhino cow and calf feeding on Euphorbia, in Damaraland, Namibia. With its large horns, a mature individual like this is a prime target for poachers. The calf of the slaughtered mother would simply be left to die; photograph: Tom Collier.

Rhinos are currently experiencing unprecedented poaching pressure (Fig. 1), with rates of one animal killed every 13 hours in some areas, and are fast heading towards wholesale extinction in the wild (Biggs *et al*. 2013). In fact, following a precipitous, poaching‐induced population crash in the 1960s (Emslie & Brooks 1999), the African western black rhino was declared extinct by the International Union for Conservation of Nature (IUCN) in 2011 (Biggs *et al*. 2013). As the price of ivory is rising, elephants fare little better and could be virtually extinct across most of their African range by 2020, unless poaching off‐take is considerably reduced (Wasser *et al*. 2008; see also Maisels *et al*. 2013). Finally, tigers are another group under extreme pressure (Nowell & Xu 2007; Walston *et al*. 2010), with three subspecies having already been lost in the last 70 years, and a lack of confirmed sightings from southern China likely signalling another extinction event (Tilson, Traylor‐Holzer & Jiang 1997).

## Mission impossible?

Many anti‐poaching measures have been explored over the years (Sutherland 2008), including the following: environmental education programmes, to reduce demand for wildlife parts in East Asia (Lee & Tilbury 1998; Nowell & Xu 2007); legalization of high‐value products, such as ivory or rhino horn, to control trade dynamics (Gillson & Lindsay 2003; Martin *et al*. 2012; Biggs *et al*. 2013); targeted monitoring of money‐laundering activities, to hamper illegal trading (as highlighted by a recent international summit; Coghlan 2014); drastic *in situ* management of threatened animal populations, such as large‐scale dehorning of rhinos, to reduce poaching opportunities (Lindsey & Taylor 2011); and ‘militarization’ of nature reserves (Milliken & Shaw 2012; see below), to facilitate arrests and deter criminal activities. As we have illustrated above, however, illegal trade in wildlife products remains rife, and novel solutions are urgently needed.

Our proposal aims at increasing the effectiveness of a widely used approach for protecting the most critically endangered species, the deployment of mobile, armed anti‐poaching units (Milliken & Shaw 2012). While these teams are often highly trained and well equipped, they generally have no way of knowing the exact time and location of poaching events. Since many target species are wide ranging and live in inaccessible habitats, this means that carcasses are often only found days or weeks after death (Martin 2001). As a result, arrests of poachers are rare and resources are mainly being focussed on securing evidence (Wasser *et al*. 2008), which is often insufficient for successful prosecution. Our proposed real‐time poaching‐alert systems would enable rangers to head towards crime scenes with rapid response times, substantially increasing the chances of apprehending suspects. In conjunction with legislation that ensures the severe punishment of convicted poachers, these altered risk dynamics should substantially reduce the economic attractiveness of poaching, giving heavily persecuted animal populations time to recover. In fact, even a temporary slowing of harvest rates would be valuable, as it would allow longer‐term measures – such as educational programmes – to deliver benefits.

## Smart electronics

**Figure 2:**
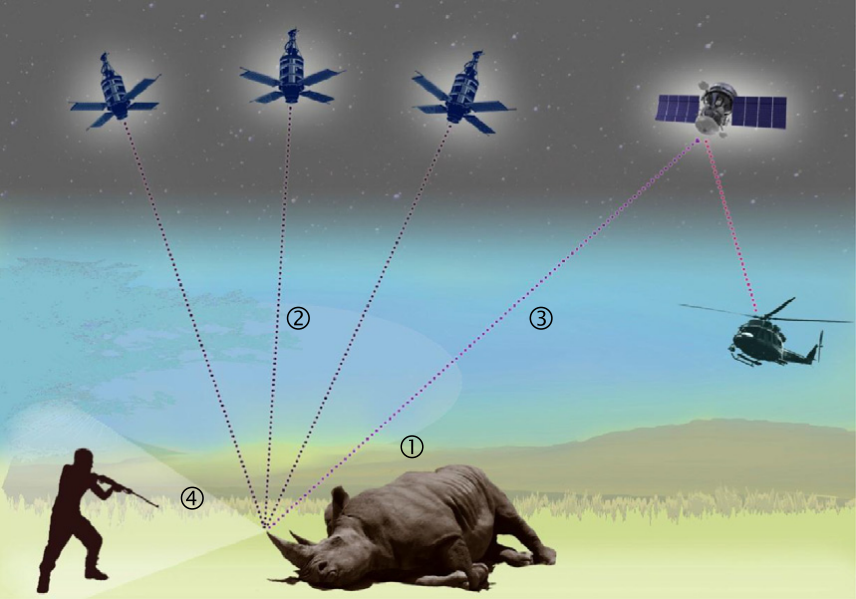
Schematic illustration of the proposed real‐time poaching‐alert system. An electronic tag is fitted inside a rhino's horn (cf. Fig. 1). Multiple sensors continuously monitor the behaviour and physiology of the tagged animal, detecting when it is shot or otherwise badly injured [①]. Once a poaching event has been recorded, a GPS unit boots up to establish the exact location of the animal [②]. Information about the event is then transmitted via satellite uplink [③] to an anti‐poaching team that heads towards the crime scene by helicopter, in an effort to intercept the poacher(s). Meanwhile, after raising the alert, the horn‐mounted tag triggers a miniature camera, which transmits video evidence [④] until the rangers arrive. Graphic: Steve Thompson (http://stevethompsondesign.com/).

The rationale of our proposed biologging system is straightforward (for a schematic illustration, see Fig. 2, and for a summary of key challenges, see Table 1). Animals are fitted with miniature electronic tags that detect poaching events and transmit relevant information remotely to anti‐poaching units on the ground. In terms of technological implementation, the integration of a few existing, well‐tested components would enable an effective three‐step process for raising an alarm: detection –location – transmission/alert. Exact system specifications will depend on a wide range of factors, including the size, behaviour and habitat preferences of the species in question, as well as the availability of local infrastructure and other resources, but the following description outlines key principles.

**Table 1:** Key challenges for developing real‐time poaching‐alert systems. See main text for possible solutions to some of these problems

(a) Technological challenges	
Poaching sensor	Sensors must trigger reliably, which requires extensive pre‐deployment testing; sensors must trigger quickly – detecting lack of motion alone (e.g. with old‐fashioned ‘jitter’ mortality switches) is insufficient, because of unacceptable time delays (see main text); some sensors (e.g. heart‐rate sensors) would require invasive procedures, such as (electrode) implantation, with possible effects on subjects’ welfare and on tagging speed (see below)
*ad hoc* data generation and transmission	Tags must generate (GPS) coordinate information and transmit alerts to satellites and/or ground receivers, before they can be destroyed by poachers; bandwidth is likely to be an issue and will necessitate data compression; where mobile phone networks are not available, dedicated infrastructure may need to be set up
Battery power	Tags’ batteries should last as long as possible, to minimize the need for retrapping subjects (see below)
Tag attachment	Tags must be attached to animals in a way that they are well concealed and achieve reliable sensor readings, without causing undue burden; invasive procedures (see above) will increase handling time, potentially hampering efforts of mass deployment (see below)
(b) Other challenges	
Permits for deployment	Some drone‐based projects experienced problems with obtaining permits for deployment; support of local authorities, and other stakeholder groups, is required
System costs	System costs should be minimized, to facilitate mass deployment
Trapping effort	A large proportion of animals must be (perceived to be) tagged, for establishing a successful deterrent function; this may be possible in small, extensively managed populations, but would be difficult in vast patrol areas; efforts of mass deployment would benefit from low system costs (see above) and straightforward deployment techniques (see above)
Infrastructure requirements	Anti‐poaching units must be able to reach remote crime scenes quickly, once an alert has been raised by a system; this will usually require the use of helicopters
Sentencing of apprehended poachers	Real‐time poaching‐alert systems can only become a major deterrent if they increase the chances of arresting poachers, and if arrests lead to successful prosecution and appropriate sentencing; local authorities need to ensure the latter

A range of sensors could be used to detect when an animal is shot or trapped, including accelerometers or heart‐rate sensors (Rutz & Hays 2009; see Table 1). To avoid false alarms, sensors would require careful calibration before system deployment and could even be combined within a single tag, to enable redundant event‐triggering (i.e. multiple sensors must trigger before the tag raises an alarm) or remote validation – for example, an accelerometer could trigger an integrated video camera (Fig. 2; Rutz *et al*. 2007; Watanabe & Takahashi 2013) or microphone (Lynch *et al*. 2013). Once the tag's sensors have confirmed a poaching event, an on‐board GPS receiver is booted up (Tomkiewicz *et al*. 2010), to establish the position of the trapped, injured or dead animal. State‐of‐the‐art systems can estimate coordinates of suitable accuracy (within tens of metres) within split‐seconds, with minimal power requirements (e.g. Fastloc). In the final step, the tag communicates the event – that is, animal ID, trigger time, sensor readings and GPS coordinate information – to a mission control centre and/or directly to rangers in the field. This could be achieved through various routes, including satellite uplinks (e.g. Iridium), UHF transmission, or pre‐existing or *ad hoc* mobile phone networks. We estimate that a well‐designed system could raise an alarm within *ca*. 10 s, which in the majority of scenarios will be faster than poachers could reach the animal and destroy its tag. Anti‐poaching units often have helicopters at their disposal, ensuring that crime scenes could be reached within minutes, or tens of minutes, after receiving an alert (Fig. 2), even in vast and inaccessible patrol areas. Where helicopters are not available, reserves would at least be warned of ongoing poaching activity, enabling them to focus ranger resources spatially, patrol park perimeters and conduct targeted vehicle checks, greatly increasing the chances of apprehending poachers.

The proposed technology should not be confused with standard satellite tracking, as routinely used with endangered species (e.g. Galanti *et al*. 2006). Although conventional GPS loggers could in principle be employed to infer poaching events from animals’ movement trajectories, costly time delays – to establish whether a stationary animal is merely resting or has indeed been injured or killed – would rule out their utility for guiding *ad hoc* intervention. Furthermore, constant sampling and relaying of positional data would quickly deplete batteries (in cases where solar power is not an option), which is not an issue with the ‘one‐shot’ tags we envisage here. Likewise, the marking of animals with PIT/RFID chips (Casey 2014), or with cutting‐edge life‐history tags (e.g. Horning & Mellish 2012), only enables the *post hoc* identification of mortalities, but cannot support a real‐time response, which lies at the heart of our proposal (for the use of real‐time ‘listening’ stations, to detect illegal logging, see Gross 2014).

We can think of many ways to tailor system specifications to suit particular species or deployment contexts, or to extend basic system functionality. For example, event‐triggering could be combined very effectively with another anti‐poaching technology that is currently being developed – unmanned aerial systems, or ‘drones’ (Marks 2013, 2014; Casey 2014; Gross 2014; Mulero‐Pázmány *et al*. 2014). Rather than putting (tagged) animals under intermittent or constant drone surveillance, however, as currently planned, poaching‐alert tags could guide drones selectively to confirmed crime scenes, for collection of still‐image or video evidence until anti‐poaching units arrive on the ground. Such targeted monitoring should considerably increase the effectiveness of drone‐based projects, while reducing their logistical complexity and running costs.

## Practical considerations

It is useful to explore briefly the practicalities of implementing our approach (cf. Table 1). Assuming that the engineering challenges of constructing suitable tags can be met, a key requirement is adequate tagging effort. Our approach aims at escalating the potential risks involved in committing poaching crimes, driving an unfavourable cost‐benefit ratio for poachers. This can only be achieved if a substantial proportion of local animal populations is marked with poaching‐alert tags or is at least being perceived to be marked, forcing poachers to take an increased risk, every time they pull the trigger or check a snare. It would of course be desirable if tags were difficult to see at a distance, because they are either very small or well hidden (e.g. in the horn of rhinos, or in ankle bracelets that cannot be seen in high grass; see Fig. 1), but where this is impossible (e.g. because tags need to be mounted on a collar, as with tigers), the strategic use of cheap dummy tags could considerably reduce programme costs (dummy tags are often used in biologging projects, to assess tagging effects; e.g. Bridger & Booth 2003). Trapping effort would admittedly pose significant challenges for large populations, but is unlikely to be an issue in those areas where intervention is most urgently needed: this is because critically endangered populations are often heavily managed, with large numbers of subjects being routinely trapped for ID marking (Ngene *et al*. 2011) and health checks.

As with any new technology employed in antagonistic contexts, one particular concern is the possible development of counter measures. In our case, this could involve, for example, technology to jam tags’ two‐way communication with satellites. We think that such an ‘arms race’ is unlikely, at least in the short term, given the required levels of technological expertise, and the substantial costs involved, which would quickly diminish criminals’ profit margins.

## Quick action

For two main reasons, we are surprised that real‐time poaching‐alert systems have not been implemented yet. First, the fight against most other types of crime heavily relies on the use of event‐triggered technology. While large‐scale CCTV surveillance, and regular police patrols, may lead to reductions in crime rates (e.g. Levitt 1997), the success of policing is no doubt dramatically enhanced by systems that raise alarms in real‐time and enable arrests at crime scenes. This includes house and car alarms, panic buttons and rape alarms, and perhaps most importantly, the victims’ ability in many circumstances to phone the police directly. We see no reason why this powerful route of ‘self‐reporting’ could not be emulated in the desperate fight against poaching crime. To our knowledge, this opportunity has so far been overlooked, despite increasing interest in technology‐driven approaches (see above). Secondly, over the last 10 years or so, significant advances have been made in biologging science, producing tags of unprecedented miniaturization, sophistication and integration (Rutz & Hays 2009) – while major engineering challenges lie ahead (see Table 1), the construction of real‐time poaching‐alert systems is well within reach of current expertise.

We hope others will join us in our efforts to implement the ideas outlined in this essay. To start with, we invite biologging engineers – many of whom already have keen interests in conservation biology (Cooke 2008; Bograd *et al*. 2010) – to collaborate with us on system development, as free sharing of expertise and other resources will be essential to making rapid progress. But, success will also depend on support from wildlife biologists and ranger teams on the ground, and on the willingness of governments and other authorities to issue permits for system deployment, to facilitate the cross‐border pursuit of criminal suspects and to put in place robust legislation for the sentencing of convicted poachers (cf. Maisels *et al*. 2013; see Table 1). Given that many target species are fast heading towards extinction, we need to explore all available anti‐poaching tools with utmost urgency, aiming for intervention at every stage of the trade chain. While we are fully aware that our reactive, technology‐based approach does not provide an all‐encompassing solution, it should – through its contribution to improving arrest rates and establishing an effective deterrent – buy crucial time until longer‐term, preventive measures have gained sufficient traction.

## Author contributions

PO initiated this collaboration; PO and CR conceived ideas; PO and CR conducted research; and CR drafted the manuscript, which was edited by PO and CR.

## Competing interests

The authors have started developing prototype poaching‐alert tags, but do not intend to exploit the technology commercially.

## Data accessibility

Data have not been archived because this article does not contain data.

## Biosketch

**Paul O'Donoghue** is an applied ecologist with a PhD from the University of Sheffield. Through his work on black rhinos in Namibia and South Africa, he has gained considerable ‘front‐line’ experience of fighting poaching crime. **Christian Rutz** is an evolutionary ecologist with a DPhil from the University of Oxford. He uses cutting‐edge ‘biologging’ technologies extensively in his field projects and has pioneered the use of miniature video cameras and proximity loggers for studying wild birds. By pooling their diverse practical expertise, Paul and Christian hope to make a contribution to the development of innovative real‐time anti‐poaching tools.
